# Complex phenotype with social communication disorder caused by mosaic supernumerary ring chromosome 19p

**DOI:** 10.1186/s12881-014-0132-3

**Published:** 2014-12-11

**Authors:** Caroline Demily, Massimiliano Rossi, Gabrielle Chesnoy-Servanin, Brice Martin, Alice Poisson, Damien Sanlaville, Patrick Edery

**Affiliations:** Centre de dépistage et de prises en charge des troubles psychiatriques d’origine génétique, Pôle Ouest, Centre Hospitalier le Vinatier, 95 bld Pinel, 69677 Bron cedex, France; Centre de Neuroscience Cognitive, UMR 5229 (CNRS et Université Lyon 1), Lyon, France; Hospices Civils de Lyon, service de génétique et centre de référence des anomalies du développement, GHE, Lyon, France; Centre de Recherche en Neurosciences de Lyon, Inserm U1028, UMR CNRS 5292, Université Claude Bernard Lyon 1, Lyon, France; Service Universitaire de Réhabilitation, Centre Hospitalier le Vinatier, Bron, France; Hospices Civils de Lyon, service de génétique, centre de référence des anomalies du développement, laboratoire de cytogénétique, GHE, Lyon, France

**Keywords:** Genetics, Autism, Social communication disorder, Duplication, Neurodevelopment, Chromosomal abnormalities, Trisomy, Copy number variants

## Abstract

**Background:**

Deletions or duplications of chromosome 19 are rare and there is no previous report in the literature of a ring chromosome derived from proximal 19p. Copy Number Variants (CNVs) responsible for complex phenotypes with Social Communication Disorder (SCD), may contribute to improve knowledge about the distinction between intellectual deficiency and autism spectrum disorders.

**Case presentation:**

We report the clinical and cytogenetic characterization of a patient (male, 33 years-old, first child of healthy Portuguese non-consanguineous parents) presenting with a complex phenotype including SCD without intellectual deficiency and carrying a mosaic supernumerary ring chromosome 19p. Microarray-Based Comparative Genomic Hybridization and Fluorescence in situ Hybridization were performed. Genetic analysis showed a large mosaic interstitial duplication 19p13.12p12 of the short arm of chromosome 19, spanning 8.35 Mb. Our data suggested a putative association between psychosocial dysfunction and mosaic pure trisomy 19p13.2p12.

**Conclusion:**

This clinical report demonstrated the need to analyze more discreet trait-based subsets of complex phenotypes to improve the ability to detect genetic effects. To address this question and the broader issue of deciphering the yet unknown genetic contributors to complex phenotype with SCD, we suggest performing systematic psychological and psychiatric assessments in patients with chromosomal abnormalities.

## Background

Deletions or duplications of chromosome 19 are rare and there is no previous report in the literature of a ring chromosome derived from proximal 19p. We report the clinical and cytogenetic characterization of a patient presenting with several abnormalities including Social Communication Disorder (SCD) without Intellectual Deficiency. This patient presented a complex phenotype with neurocognitive features, dysmorphism, growth delay and SCD. He was carrying a mosaic pure trisomy 19p13.2p12.

Pathogenic copy number variants (CNVs) are found in nearly 20% of individuals with Intellectual Deficiency (ID) [1] and in 10% of patients showing Autism Spectrum Disorders (ASD) [2]. Nevertheless, the relevance of making a clinical distinction between ID and ASD in terms of genetic etiology remains controversial because of considerable overlaps of the causative genes or chromosomal regions. Copy Number Variants (CNVs) responsible for complex phenotype with Social Communication Disorder (SCD), a diagnosis related to ASD, may contribute to improve knowledge about this data.

## Case presentation

### Clinical report

The patient was the first child of healthy Portuguese non-consanguineous parents. The father’s height was 163 cm and the mother’s was 148 cm. Family history was otherwise unremarkable. Pregnancy and delivery were normal: birth weight was 2.550 kg, at −2 standard deviations (SD); birth length was 46 cm (−3 SD); head circumference: 32 cm (−3SD), Apgar score was 10, at 1 and 5 min. Short stature of prenatal onset was noted.

During infancy, he showed gastro-esophageal reflux and recurrent otitis media. He underwent surgical interventions for inguinal hernias, adenoidectomy and umbilical cyst ablation.

He had mild motor delay and started walking at the age of 2 years. He subsequently showed moderate learning difficulties. He has undergone several trainings in electricity and computing, but to no avail. He has had various odd jobs (e.g. waiter, fast food employee) and currently lives on his own.

**Figure 1:**
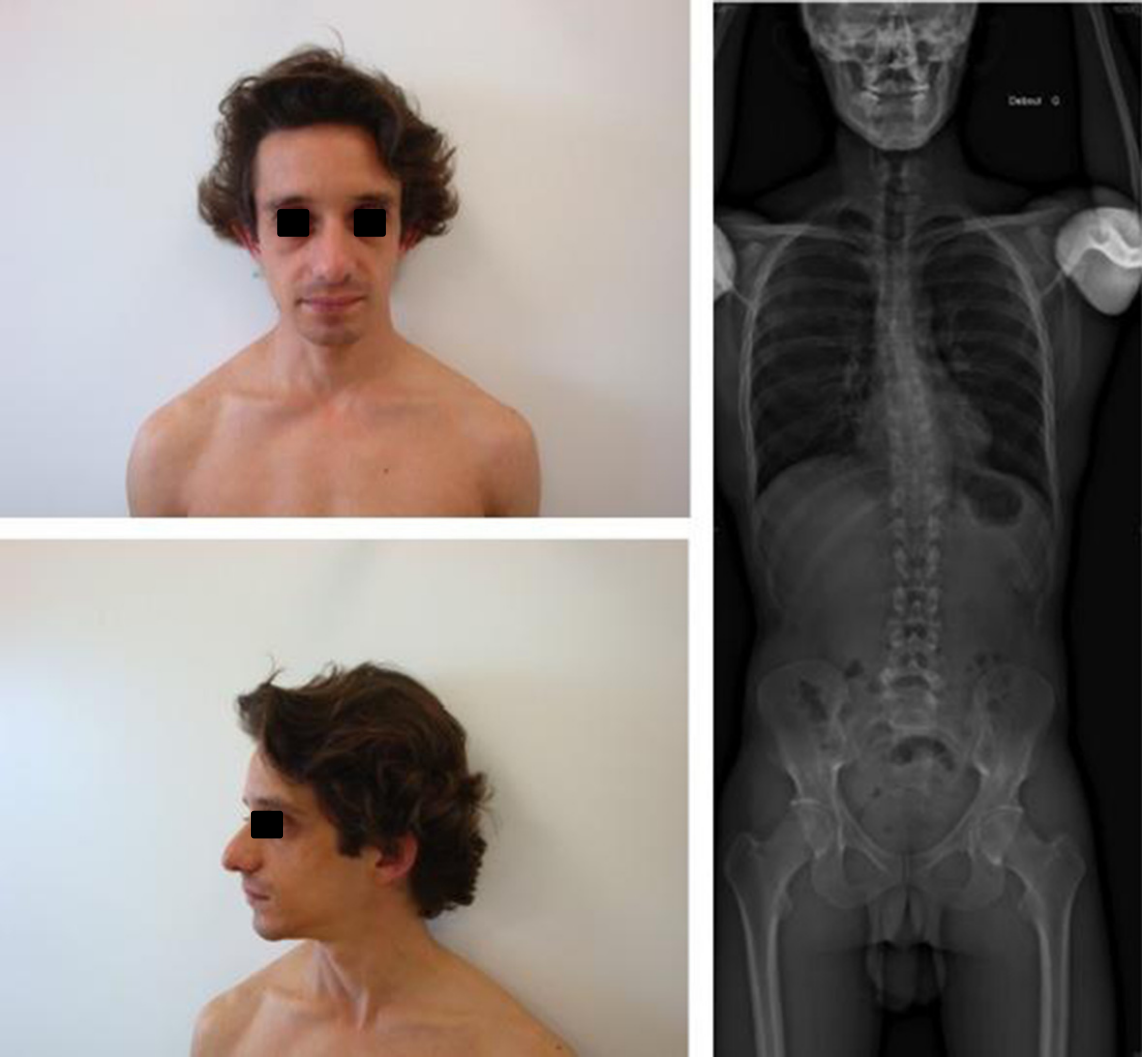
**Facial dysmorphism of patient (a face and b profile) and X-Rays showing the scoliosis (c).**

He was referred to our department at the age of 33 years. Clinical data showed that he was 152 cm tall (−3,5SD) and weighed 43.9 kg; body mass index was normal (18.9 kg/m^2^) as well as head circumference (55 cm). He had a long face, high forehead, thick eyebrows, down-slanting palpebral fissures, a prominent nose with high nasal bridge and malar hypoplasia (Figure 1a and b). He had a mild scoliosis (Figure 1c) and neurological examination was normal.

He presented a psychiatric phenotype. He presented difficulties in acquiring/using language, limited effective communication and social relationships. A social communication disorder was diagnosed according to DSM-5 criteria. The neuropsychological evaluation documented a normal intellectual functioning (total IQ: 90). The patient showed decreased psychomotor speed impacting on attentional tasks and mildly impaired verbal memory. However, he had good executive functioning and visual memory abilities.

Complete diagnostic assessment, including fragile X molecular analysis, full blood count, ammonia, plasma amino acids, urine orotic acid, screening for creatine metabolism deficiencies and urinary organic acids, was normal. Magnetic resonance imaging of the brain was normal. Skeletal survey showed mild scoliosis with no obvious sign of bone dysplasia. Echocardiography was normal and abdominal ultrasounds scan showed isolated mild hepatomegaly; liver function tests were normal.

### Cytogenetic analysis

Genomic analyses were performed after obtaining a signed informed consent, according to French legislation. Those analyses are performed routinely and do not need specific ethical approval by a committee.

#### Chromosome analysis

Conventional blood lymphocytes karyotypes (both GTG and RHG-banding) were performed according to standard methods.

#### Microarray-Based Comparative Genomic Hybridization (aCGH)

Genomic DNA extraction and aCGH were performed as previously described, with an 180,000-oligonucleotide (180 K) microarray (Sure Print G3 Human CGH Microarray Kit, Agilent Technologies, Santa Clara, CA) [3]. The presence of a copy number variation was considered when at least three contiguous oligonucleotides showed an abnormal log2 ratio. Array-CGH results were analyzed using the UCSC hg19 assembly. The average gain of log2 ratio was calculated, for each dye-swap experiment (two results for each patient) and the level of mosaicism was also calculated [4].

#### Fluorescence In Situ Hybridization (FISH)

FISH was performed with the BAC clone CTD-2332E1 located in 19p12 (chr19:20,794,365-20,798,195 bp, hg19). The probe was FITC-labelled by nick-translation, as previously described [3] and hybridized on metaphase spread, together with the 19qter control probe (Cytocell, Cambridge, UK).

### Results

**Figure 2:**
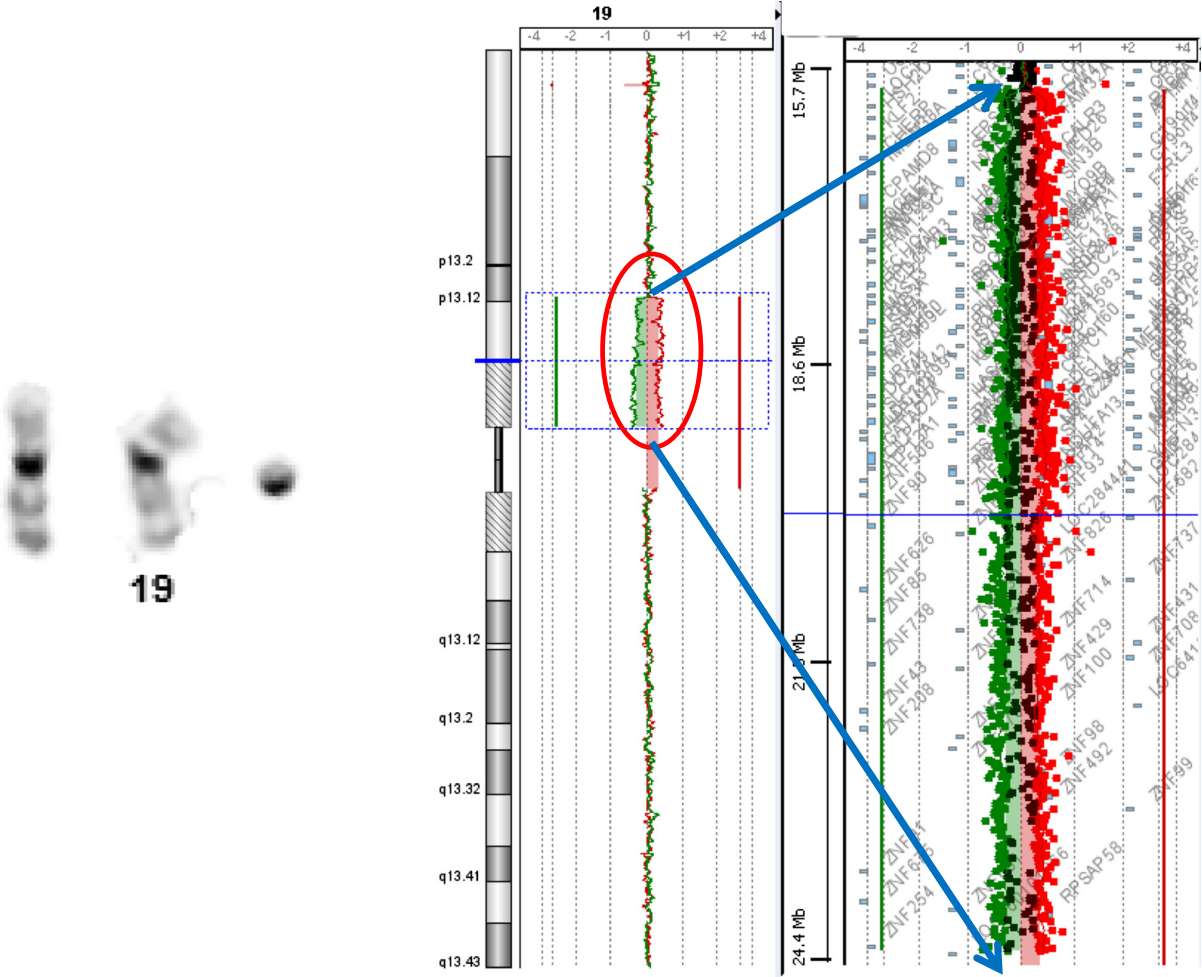
**Partial GTG karyogramme showing the r(19) and chromosome 19 aCGH profile showing a genomic gain spanning 8.35 Mb.**

Blood standard karyotype showed the presence of a small supernumerary marker chromosome (sSMCs). This marker was present in 72% of examined cells (18/25). aCGH showed a large mosaic interstitial duplication of the short arm of chromosome 19, spanning 8.35 Mb :arr[hg19] 19p13.12p12(15,987,511-24,340,741)x2 ~ 3 (Figure 2). The level of mosaicism was evaluated at 55%.

FISH confirmed the presence of the sSMCS in 50% of examined cells (40/70 metaphase cells and 44/100 nucleic cells) and allowed us to conclude that this sSMCs was a ring of chromosome 19 encompassing only a part of the 19p genomic region. FISH analysis, performed in both parents, was normal, thus confirming the *de novo* origin of the ring chromosome 19 observed in the patient.

### Discussion

Analysis of genomic rearrangements using aCGH in patients with various symptoms including psychiatric features exposes unexpected complexity. First of all, social communication disorder in people with chromosomal abnormalities may be much more common than reported because psychological or psychiatric assessments are not systematically carried out.

Moreover, molecular studies are usually performed for ID, ASD and/or syndromes of multiple congenital abnormalities. The genetic background of ASD is highly heterogeneous and the fact of having most common or rare CNVs may usually not be considered as a unique cause, but may occasionally increase the risk of developing ASD. Recently, Pinto et al. [5], showed that rare CNVs are an important source of risk for ASD. Also, a genome-wide screen for autism loci identified the best compatibility with linkage to 17q11.2 and 19p13, with maximum multipoint heterogeneity LOD scores of 2.9 and 2.6, respectively [6]. The mosaic gain identified with aCGH in our patient encompasses 398 genes including 97 OMIM genes and 13 morbid OMIM genes, namely *CRFL1*, *RFXANK*, *IL2RB1*, *MYO9B*, *JAK3*, *SLC5A5*, *COM*, *GDF1*, *GTPBP13*, *NDUFA13*, *INSL3*, *PIK3R2*, and *CALR3*.

To our knowledge, only 9 cases with extra ring 19chr were reported [7]. The short arm of chromosome 19 only was involved using either FISH or aCGH in two of these cases. Both patients had cerebral abnormalities including respectively enlarged cerebral ventricles and cortical atrophy [8] and Dandy–Walker malformation [8]. Among these cases, only case number 19-W-p12/2-1 is suitable for comparison, because the sSMC was studied by aCGH and the genomic region included 2.53 Mb of the 19p pericentromeric region. This patient showed hip subluxation, pes calcaneovalgus congenitus, periodic breathing, congenital stridor and feeding problems. At 3 months of age, an extreme restlessness, nearly opisthotonos and at 5 months of age hyperexcitability, and developmental delay were noted. Unfortunately, social communication was not described. In Liehr’s database (http://ssmc-tl.com/sSMC.html), 43 sSMC derived from the chromosome 19 were reported and 70% have clinical features. The polymorphic region proposed by Liehr spanned from 15.2 Mb to 39.08 Mb genomic positions, thus including the duplicated genomic region identified here. However, social cognitive aspects of individuals considered as asymptomatic with a CNV of this genomic region were not studied in detail.

## Conclusion

This clinical report suggests 19p13.12p12 as a possible SCD susceptibility locus and demonstrates the need to analyze more discreet trait-based subsets of complex phenotypes to improve the ability to detect genetic effects. To address this question and the broader issue of deciphering the yet unknown genetic contributors to complex phenotype with SCD, we suggest performing psychological and psychiatric assessments in patients with chromosomal abnormalities.

## Consent

Written informed consent was obtained from the patient for publication of their individual details and accompanying images in this manuscript. The consent form is held in the patients’ clinical notes and is available for review by the Editor-in-Chief.

## Abbreviations

SCD: Social communication disorder
ASD: Autism spectrum disorder
CNVs: Copy number variants
ID: Intellectual deficiency
DSM-5: Diagnostic and statistical manual – Fifth revision
SD: Standard Deviation
FISH: Fluorescence In Situ Hybridization
aCGH: Microarray-based comparative genomic hybridization
